# Comprehensive in vitro and in ovo assessment of cytotoxicity: Unraveling the impact of sodium fluoride, xylitol, and their synergistic associations in dental products

**DOI:** 10.17305/bb.2024.10181

**Published:** 2024-08-01

**Authors:** Daniel Breban-Schwarzkopf, Raul Chioibas, Ioana Macasoi, Sorin Bolintineanu, Iasmina Marcovici, George Draghici, Stefania Dinu, Roxana Buzatu, Cristina Dehelean, Camelia Szuhanek

**Affiliations:** 1 Faculty of Medicine, “Victor Babes” University of Medicine and Pharmacy Timisoara, Timisoara, Romania; 2 Faculty of Pharmacy, “Victor Babes” University of Medicine and Pharmacy Timisoara, Timisoara, Romania; 3 Research Center for Pharmaco-Toxicological Evaluations, Faculty of Pharmacy, “Victor Babes” University of Medicine and Pharmacy Timisoara, Timisoara, Romania; 4 Faculty of Dental Medicine, “Victor Babes” University of Medicine and Pharmacy Timisoara, Timisoara, Romania; 5 Pediatric Dentistry Research Center, Faculty of Dental Medicine, “Victor Babes” University of Medicine and Pharmacy Timisoara, Timisoara, Romania; 6 Orthodontic Research Center (ORTHO-CENTER), “Victor Babes” University of Medicine and Pharmacy Timisoara, Timisoara, Romania

**Keywords:** Natrium fluoride (NaF), xylitol (Xyl), safety considerations, caspase activity, pro-apoptotic gene expression, hen’s egg test-chorioallantoic membrane (HET-CAM) assay

## Abstract

Over the past several decades, dental health products containing fluoride have been widely employed to mitigate tooth decay and promote oral hygiene. However, concerns regarding the potential toxicological repercussions of fluoride exposure have incited continuous scientific inquiry. The current study investigated the cytotoxicity of sodium fluoride (NaF) and xylitol (Xyl), both individually and in combination, utilizing human keratinocyte (HaCaT) and osteosarcoma (SAOS-2) cell lines. In HaCaT cells, NaF decreased proliferation in a concentration-dependent manner and induced apoptosis-related morphological changes at low concentrations, whereas Xyl exhibited dose-dependent cytotoxic effects. The combination of NaF and Xyl reduced cell viability, particularly at higher concentrations, accompanied by apoptosis-like morphological alterations. Sub-cytotoxic NaF concentrations (0.2%) significantly affected caspase activity and the expression of pro-apoptotic genes. Conversely, Xyl demonstrated no discernible effect on these biological parameters. In SAOS-2 cells, NaF increased proliferation at high concentrations, contrasting with Xyl’s concentration-dependent cytotoxic effects. The combination of NaF and Xyl had a minimal impact on cell viability. Sub-cytotoxic NaF concentrations did not influence caspase activity or gene expression, while Xyl induced dose-dependent morphological alterations, increased caspase activity, and upregulated pro-apoptotic gene expression. In ovo experiments on the chorioallantoic membrane (CAM) revealed that only NaF induced irritant effects, suggesting potential vascular adverse outcomes. This study advocates for the combined use of NaF and Xyl, highlighting their cytotoxicity benefits in healthy cells while maintaining safety considerations for tumor cells.

## Introduction

The global prevalence of dental caries, colloquially known as tooth decay or cavities, persists as a prominent and consequential concern within the realm of public health [1]. Dental caries stands as one of the most pervasive chronic ailments, afflicting individuals across the age spectrum, with a particular proclivity for children, adolescents, and the elderly. This incidence arises from a complex interplay of factors, predominantly encompassing the dynamic interactions between oral microorganisms, fermentable carbohydrates, and susceptible dental surfaces [2]. Compounded by inadequate oral hygiene practices, a diet characterized by high sugar content, and socio-economic determinants, the predisposition to dental caries is heightened. Despite advancements in preventive strategies, such as the introduction of fluoridated water, dental sealants, and enhanced oral health education, dental caries continue to affect a considerable segment of the population, giving rise to discomfort and pain, and imposing substantial economic burdens on healthcare systems [3]. A vigilant approach to oral care, routine dental examinations, and comprehensive public health initiatives must persist as pivotal endeavors in mitigating the incidence of dental caries while fostering overall oral health [4].

The relationship between dental caries and oral carcinogenesis, particularly bone neoplasms, has been rigorously examined in the scientific literature. Oral cancer etiology is indirectly influenced by dental caries through mechanisms involving chronic inflammation, oral mucosal disruption, changes in the oral microbiome, and long-term exposure to potential carcinogens [4, 5]. While the direct causal inferences to bone malignancies within the oral cavity remain infrequent, the shared behavioral risk factors, such as tobacco use and alcohol consumption, highlight the importance of early dental intervention and the adoption of a health-promoting lifestyle. These measures are crucial for mitigating the collective risks associated with dental caries and oral cancer [6–8].

To preserve dental health and prevent dental disease, sodium fluoride (NaF) is incorporated into toothpaste formulations. The recognition of its prominence in dental care is attributed to its effectiveness in controlling dental caries, strengthening dental enamel, and improving overall oral hygiene [9]. NaF plays a pivotal and scientifically well-established role in dental caries prevention, operating through a multifaceted mechanism [10]. When introduced into the oral milieu, it interacts with the hydroxyapatite in tooth enamel to form fluorapatite, a highly resilient compound that exhibits high resistance to acid-mediated demineralization, a process mainly caused by the metabolism of dietary sugars by oral bacteria [11]. Moreover, NaF demonstrates efficacy in the inhibition of metabolic activities of acid-producing oral bacteria, notably *Streptococcus mutans*, thereby diminishing their acidogenic potential. Complementary to these actions, NaF also exerts antibacterial effects that hinder bacterial virulence and adhesion processes, consequently mitigating the formation of dental plaque, a biofilm intricately linked with caries pathogenesis [12].

Xylitol (Xyl), an endogenous sugar alcohol, has garnered attention in dental hygiene formulations due to its distinctive attributes and potential advantages for oral health [13]. In recognition of its non-cariogenic properties, Xyl has been incorporated into various dental products, such as toothpaste, where it has demonstrated effectiveness in preventing the proliferation of cariogenic bacteria, especially *Streptococcus mutans* [14]. Its antimicrobial activity is ascribed to Xyl’s disruption of bacterial metabolism and its inability to function as a substrate for acid-producing bacteria, thereby diminishing the susceptibility to dental caries [14]. Within the context of dental preparations containing NaF, the interaction between Xyl and fluoride necessitates meticulous scrutiny. Although NaF is established for its capacity to fortify tooth enamel and forestall cavities, the potential synergistic or antagonistic effects arising from its combination with Xyl require thorough investigation. Grasping the intricate interplay between these substances is imperative for the refinement of dental formulations, ensuring efficacy in caries prevention while mitigating any inadvertent cytotoxic implications. This inquiry assumes paramount importance in influencing the trajectory of dental care products, striving to advance oral health outcomes while upholding a commitment to overall safety [15–17].

Based on these principles, the current study was designed to systematically assess the in vitro and in ovo effects resulting from the exposure to NaF, Xyl, and their combinations. To this end, the study comprehensively evaluated various parameters including cell viability and morphology, effects on nuclei and actin filaments, as well as the enzymatic activities of caspases 3/7 and 9. It also explored the expression levels of anti-apoptotic and pro-apoptotic genes within an in vitro setting. Additionally, the study examined the irritative effects of these substances on the chorioallantoic membrane (CAM).

## Materials and methods

### Reagents

This study was conducted using a range of reagents, which were sourced as follows: NaF, Xyl, phosphate-buffered saline (PBS), fetal calf serum (FCS), penicillin-streptomycin, 3-(4,5-dimethylthiazol2-yl)-2,5-diphenyltetrazolium bromide (MTT) were acquired from Sigma Aldrich Merck KgaA (Darmstadt, Germany); B-cell lymphoma 2 (Bcl-2) protein, Bcl-2-associated X protein (Bax), Maxima^®^ First Strand complementary DNA (cDNA) Synthesis Kit, Quick-RNA™ purification kit, trizol reagent, 4,6-diamidino-2-phenylindole (DAPI), and Texas red-X Phalloidin were obtained from Thermo Fisher Scientific (Waltham, MA, United States); Bcl-2-associated death promoter (Bad) was purchased from Eurogentec (Seraing, Belgium). Dulbecco’s Modified Eagle’s Medium (DMEM) (ATCC^^®^^ 30–2002TM) and Eagle’s Minimum Essential Medium (EMEM) (ATCC^^®^^ 30-2003TM) were provided by the American Type Culture Collection (ATCC) (Lomianki, Poland). Leibovitz’s L-15 medium (P04-27500) was sourced from PanBiotech (Aidenbach, Germany). For the cytotoxicity study, the CyQUANT lactate dehydrogenase (LDH) cytotoxicity assay kit (C203000) from Invitrogen by ThermoFisher Scientific (Waltham, MA, USA) was utilized. Caspase-Glo(R) 3/7 assay (G8090) and Caspase-Glo(R) 9 assay (G8210) were purchased from Promega Corporation (Madison, WI, USA). All reagents were confirmed to possess characteristics suitable for cell culture applications.

### Cell culture

In this study, two distinct cell lines were utilized, human keratinocytes (HaCaT) and osteosarcoma cells (SAOS-2) both of which were acquired from ATCC in the form of cryopreserved vials. The maintenance of both HaCaT and SAOS-2 cell lines involved culturing them in DMEM supplemented with 10% FCS and 1% penicillin–streptomycin (penicillin at 100 U/mL and streptomycin at 100 µg/mL). During the experiment, the cells were incubated at a constant temperature of 37 ^∘^C in an atmosphere containing 5% CO_2_.

### Assessment of cellular viability

To assess the effect of the compounds on the viability of the selected cell lines, the study employed a colorimetric method, specifically the MTT assay. The experimental protocol encompassed the cultivation of cells in 96-well plates at a seeding density of 10,000 cells per well. After achieving approximately 90% confluence, the cells were subjected to a 24-h stimulation period with varying concentrations of NaF (0.05%, 0.1%, 0.2%, 0.3%, and 0.5%), Xyl (0.1%, 1%, 5%, 10%, and 20%), and a combination of NaF (0.2%) and Xyl (0.1%, 1%, 5%, 10%, and 20%). In terms of preparation of the dilutions used in the in vitro testing, ultrapure water was used to solubilize the compounds and to obtain stock concentrations. These were then further diluted with the culture medium specific to each cell line to achieve the desired concentrations.

Following the designated incubation period, 10 µL of MTT reagent was added to each well, and the cells were further incubated for 3 h at 37 ^∘^C. To dissolve the MTT formazan crystals, 100 µL of solubilizing agent was dispensed into each well, followed by a 30-min incubation period. Afterward, absorbance measurements were made at 570 nm using Cytation 5 equipment (BioTek Instruments Inc., Winooski, VT, USA). In the initial phase, the correct absorbance values were obtained by subtracting the background absorbance of the culture medium and applying the following mathematical formula:


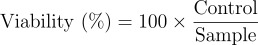


### Cytotoxicity assay

Using a methodology similar to the MTT assay, a cytotoxicity assessment was performed to determine the toxicity profile of the compounds on the selected cell lines by quantifying the LDH release into the media. For this purpose, the cells were cultivated at a density of 1 × 10^4^ cells per well, and upon reaching approximately 90% confluence, they were exposed to the test compounds. Following a 24-h incubation period with these compounds, a new plate was prepared, into which 50 µL/well of media containing the released LDH was transferred. Subsequently, a reaction mixture of 50 µL was added to each well and mixed thoroughly. The assay continued with a 30-min incubation at room temperature in the dark, which was followed by the addition of 50 µL of stop solution to each well. The absorbance was then measured using the Cytation 5 microplate reader from BioTek Instruments Inc. (Winooski, VT, USA) at wavelengths of 490 and 680 nm.

### Cellular proliferation

In order to better understand the toxicological profile of the tested compounds, the impact on cell proliferation was determined. Cells were seeded in 96-well plates at a density of 2000 cells per well, using Leibovitz’s L-15 medium for cell growth. After 24 h, the cells were exposed to concentrations of NaF, Xyl, and NaF + Xyl, which had previously been evaluated in cell viability and cellular morphology tests. The Lionheart FX automated microscope and Cytation cell imaging readers, both from BioTek Instruments Inc., were utilized for cellular proliferation determination. A bright-field image and a cell-counting image were acquired for each well every hour during the automated protocol.

### Cellular morphology evaluation

To assess the influence of NaF, Xyl, and NaF + Xyl on the cellular morphology, a microscopic examination of HaCaT and SAOS-2 cells was conducted following a 24-h exposure period. This analysis aimed to provide further insights into the potential mechanisms of action of the compounds and their interactive effects. The cells were cultured in 12-well plates at a density of 2 × 10^5^ cells per well. The morphological alterations were noted using bright field illumination facilitated by the Olympus IX73 inverted microscope (Olympus, Tokyo, Japan). Subsequently, analysis of the images was performed using CellSens Dimensions v.17 Software (Olympus, Tokyo, Japan).

### Immunofluorescence

Given the observation of significant morphological changes in the cells, the study progressed to investigate potential alterations at the level of cellular organelles important for cell survival and death. To investigate alterations in nuclear structures and actin fibers, HaCaT and SAOS-2 cells were cultured in 96-well black plates at a density of 2000 cells/well. Upon attaining approximately 90% confluence, the cells were exposed to NaF (0.2%), Xyl (5%), and a combination of NaF (0.2%) and Xyl (5%) for a 24-h duration. Immediately following the exposure, the cells were rinsed three times with ice-cold PBS and then fixed with 4% paraformaldehyde. After fixation, cells underwent permeabilization with 0.1% triton X in PBS. To mitigate the effects of 0.1% triton X, a blocking solution containing 1% bovine serum albumin (BSA) in PBS was applied. Texas red-X Phalloidin was utilized for the visualization of the actin fibers, while DAPI was incorporated for nuclei visualization. Image acquisition was performed using The Lionheart FX automated microscope from BioTek Instruments Inc. (Winooski, VT, USA). The quantification of apoptosis was conducted through the calculation of the apoptotic index (AI), employing the formula provided below [18]:


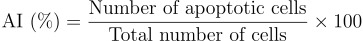


The assessment of nuclear morphology was conducted utilizing the ImageJ software, adhering to procedures outlined in the existing literature [19].

### Caspase-3/7 and 9 activity

Following the previously observed morphological changes indicative of cell apoptosis, the study proceeded to evaluate the activity of caspases, which play a major role in this cell death process. The enzymatic activities of caspases 3/7 and 9 were evaluated after subjecting HaCaT and SAOS-2 cells to a 24-h treatment with varying concentrations of NaF (0.2%), Xyl (5%), and a combination of NaF (0.2%) and Xyl (5%). The cells were cultured in 96-well plates at a density of 1 x 10^4^ per well. The Caspase-Glo assay kit, sourced from Promega (Madison, USA) was utilized for this assessment. Initially, the plates were allowed to stabilize at room temperature for the first 30 min. Subsequently, 100 µL of Caspase-Glo reagent was added to each well. This was followed by 30 s of agitation on a plate shaker and an additional incubation of 2 h at room temperature. The luminescence generated was then quantified using the Cytation 5 system by BioTek Instruments Inc. (Winooski, VT, USA).

### Gene expression

The next step of the study involved the evaluation of the expression levels of pro-apoptotic and anti-apoptotic genes, which, alongside caspases, play a major role in the cell death process. For the quantification of gene expression implicated in the cellular apoptosis process, real-time reverse transcription-polymerase chain reaction (RT-PCR) methodology was applied. The cells were cultured in 96-well plates at a density of 1 × 10^4^ cells per well. After a 24-h treatment period, the total RNA quantity was measured using a DS-11 spectrophotometer (DeNovix, Wilmington, DE, USA) in conjunction with trizol reagent and the Quick-RNA™ purification kit. The synthesis of RNA into cDNA was then conducted using the Maxima^®^ First Strand cDNA Synthesis Kit. Quantification of gene expression via RT-PCR was performed using the QuantStudio 5 RT-PCR system (Thermo Fisher Scientific, Inc., Waltham, MA, USA), together with the Power SYBR-Green PCR Master Mix.

### Hen’s egg test-chorioallantoic membrane (HET-CAM)

A HET-CAM assay was used to evaluate the irritant and toxic potential of NaF, Xyl, and their combination (NaF + Xyl) at the vascular level. For each sample, three eggs were used, in three individual experiments.

The experimental method comprised the following steps: (1) the chicken eggs (*Gallus gallus domesticus*) were disinfected using 70% ethanol, followed by incubation; (2) albumen was extracted on the fourth day of the incubation via perforation; (3) on the following day, the upper part of the eggs was drilled for the visualization of blood vessels, then sealed with adhesive tape; (4) on the 10th day of incubation, each sample (600 µL) was administered onto the CAM, including the negative control (H_2_O), positive controls (1% sodium dodecyl sulfate [SDS]) and compounds (NaF 0.2%, Xyl 5%, and NaF 0.2% + Xyl 5%); (5) as part of the observation, photographs were taken before sample addition (T0), after sample addition (T5), and at the end of the 5-min observation period (T5), documenting the compounds’ effects (hemorrhage, vascular lysis, and coagulation). This was done using the Discovery 8 stereomicroscope by Zeiss (Göttingen, Germany), equipped with a Zeiss Axio CAM 105 color camera; and (6) subsequently, the irritation score (IS) for each sample was determined using the provided formula:


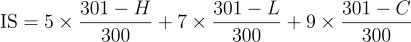


Here, IS represents the irritation score, H denotes the time of hemorrhage observation, L indicates the time of vascular lysis occurrence, and C signifies the time of intravascular coagulation establishment.

### Statistical analysis

The study outcomes were presented as means ± standard deviation (SD). Statistical differences among the compared groups were assessed using the one-way analysis of variance (ANOVA) method and Dunnett’s multiple comparison post-test. Statistical analyses were conducted using GraphPad Prism version 9.5.1 software (GraphPad Software, San Diego, CA, USA, www.graphpad.com). All tests were performed in triplicate, consisting of three independent experiments. Statistically significant differences between data are denoted with asterisks, where * represents *P* < 0.1; ** represents *P* < 0.01; *** represents *P* < 0.001; and **** represents *P* < 0.0001.

## Results

### Cellular viability evaluation

The assessment of cytotoxic potential on HaCaT and SAOS-2 cells involved testing various concentrations of NaF (at 0.05%, 0.1%, 0.2%, 0.3%, and 0.5%), Xyl (at 0.1%, 1%, 5%, 10%, and 20%), and combinations of NaF concentrations at 0.2% and Xyl concentrations at 0.1%, 1%, 5%, 10%, and 20%.

**Figure 1.**
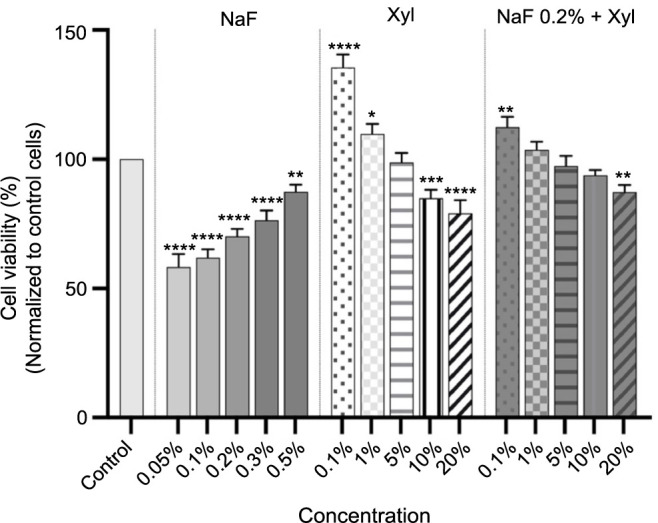
**Analyses of the cytotoxic effects of NaF at various concentrations, Xyl at various concentrations, and a combination of 0.2% NaF with various concentrations of Xyl on HaCaT cells following a 24-h treatment period.** Data are presented as percentages (%) normalized to control cells along with means ± standard deviations from three independent experiments, each performed in triplicate. One-way ANOVA and Dunnett’s multiple comparison post-test were used to analyze the significance of the differences between test and control groups. *P < 0.1; **P < 0.01; ***P < 0.001; ****P < 0.0001. NaF: Sodium fluoride; Xyl: Xylitol; HaCaT: Human keratinocytes; ANOVA: Analysis of variance.

Figure 1 illustrates the cytotoxic effects of NaF on HaCaT cells, revealing a concentration-dependent impact. Notably, the data revealed that the lowest NaF concentration of 0.05% significantly reduced cell viability, reaching a maximum decline of 58%. As concentrations increased, the cytotoxic effect lessened while remaining discernible, with cell viability approximately at 87% at the 0.5% concentration. Conversely, Xyl demonstrated an opposing effect on HaCaT cell viability. The lowest evaluated concentration of Xyl (0.1%) significantly increased cell viability to approximately 136%, and at 1%, the increase was approximately 109%. However, higher concentrations of Xyl resulted in a decreasing effect on cell viability, with no reduction going below 79%. The combined treatment of NaF at 0.2% and Xyl at concentrations of 0.1% and 1% exhibited a synergistic effect, enhancing cell viability by 100%. Even at higher concentrations, cell viability did not fall below 87%, suggesting a potential protective effect of Xyl against NaF-induced toxicity in keratinocytes (Figure 1).

A comparable pattern manifested in the context of the osteosarcoma cell line, SAOS-2. Treatment with NaF at this cellular level resulted in an augmentation in cell viability corresponding to the escalating concentration. Specifically, at the highest concentration of 0.5%, a notable increase in cell viability, approximating 124%, was documented. Concurrently, Xyl exhibited a dose-dependent reduction in cell viability. The initial two concentrations of Xyl examined did not produce a noteworthy decline in the percentage of viable cells. However, at the highest concentration tested, a marked decrease in viability to approximately 73% was recorded.

**Figure 2.**
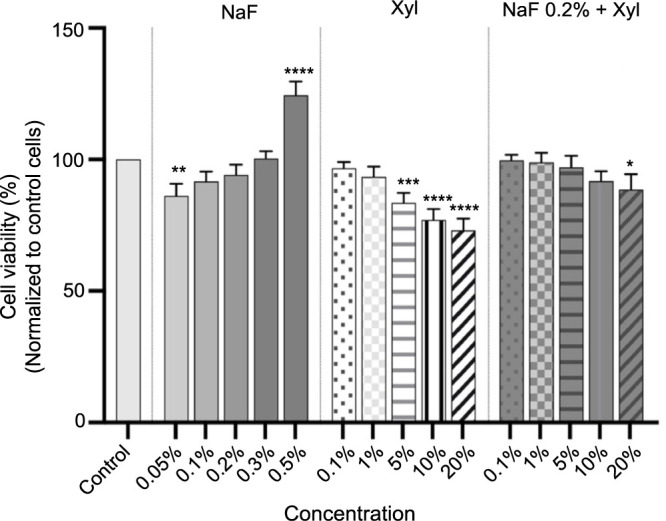
**Analyses of the cytotoxic effects of NaF at various concentrations, Xyl at various concentrations, and a combination of 0.2% NaF with various concentrations of Xyl on SAOS-2 cells following a 24-h treatment period.** Data are presented as percentages (%) normalized to control cells along with means ± standard deviations from three independent experiments, each performed in triplicate. One-way ANOVA and Dunnett’s multiple comparison post-test were used to analyze the significance of the differences between test and control groups. *P < 0.1; **P < 0.01; ***P < 0.001; ****P < 0.0001. NaF: Sodium fluoride; Xyl: Xylitol; SAOS-2: Osteosarcoma cells; ANOVA: Analysis of variance.

Regarding the combined administration of NaF and Xyl in SAOS-2 cells, substantial alterations were observed solely at the 20% Xyl concentration. At this juncture, a marginal reduction in cell viability was observed, reaching approximately 88% (Figure 2).

### Cytotoxicity assay

Upon evaluating cell viability, the study proceeded to ascertain the cytotoxic effects of the compounds using the LDH release assay.

**Figure 3.**
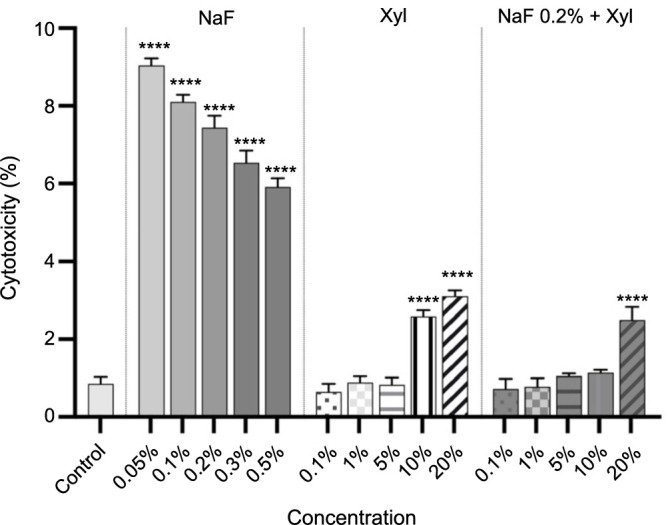
**Cytotoxicity evaluation in HaCaT cells following treatment with NaF at various concentrations, Xyl at various concentrations, and a combination of 0.2% NaF with various concentrations of Xyl, using the LDH release assay 24 h post-treatment.** Data are presented as percentages (%) normalized to control cells along with means ± standard deviations from three independent experiments, each performed in triplicate. One-way ANOVA and Dunnett’s multiple comparison post-test were used to analyze the significance of the differences between test and control groups. ****P < 0.0001. NaF: Sodium fluoride; Xyl: Xylitol; HaCaT: Human keratinocytes; LDH: Lactate dehydrogenase; ANOVA: Analysis of variance.

In HaCaT cells, NaF demonstrated a pronounced cytotoxic impact, particularly at the lowest concentrations under evaluation. Specifically, the 0.05% concentration yielded in an approximate 9% increase in cytotoxicity, while the highest tested concentration (0.5%) induced a rise in the cytotoxic percentage to approximately 6%. Regarding the cytotoxic impact of Xyl, a dose-dependent correlation was evident. Initially, the first three concentrations examined did not induce a notable cytotoxic response in keratinocytes. However, at 10% and 20% concentrations, there was an increase in cytotoxicity to 2.5% and 3%, respectively. The combined administration of NaF and Xyl resulted in the least cytotoxicity, with a marginal increase in the cytotoxic percentage of approximately 2.4% noted at the 20% Xyl concentration (Figure 3).

**Figure 4.**
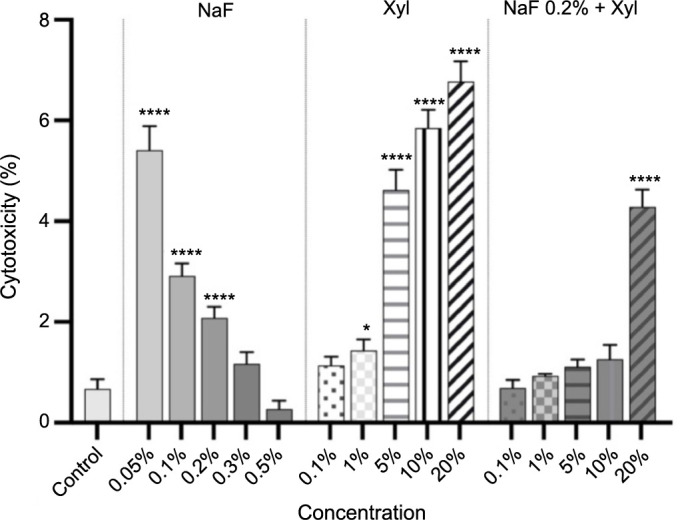
**Cytotoxicity evaluation in SAOS-2 cells following treatment with NaF at various concentrations, Xyl at various concentrations, and a combination of 0.2% NaF with various concentrations of Xyl, using the LDH release assay 24 h post-treatment.** Data are presented as percentages (%) normalized to control cells along with means ± standard deviations from three independent experiments, each performed in triplicate. One-way ANOVA and Dunnett’s multiple comparison post-test were used to analyze the significance of the differences between test and control groups. *P < 0.05; ****P < 0.0001. NaF: Sodium fluoride; Xyl: Xylitol; SAOS-2: Osteosarcoma cells; LDH: Lactate dehydrogenase; ANOVA: Analysis of variance.

In the context of SAOS-2 cells, NaF exhibited a prominent cytotoxic profile. This was particularly notable at the concentration of 0.05%, where the documented cytotoxicity percentage was approximately 5.4%. Concurrently, the concentration of 0.5% displayed the least cytotoxic effect, registering a cytotoxicity percentage of approximately 0.26%. Regarding the cytotoxic profile of Xyl in SAOS-2 cells, a dose-dependent relationship was discerned. Specifically, concentrations of 10% and 20% exerted the most substantial cytotoxic impact on tumor cells, yielding cytotoxicity percentages of approximately 5.8% and 6.7%, respectively. The combination of NaF and Xyl resulted in a noteworthy increase in the cytotoxicity percentage solely at the 20% Xyl concentration. In this instance, the cytotoxicity percentage was approximately 4.3% (Figure 4).

### Cellular proliferation

To enhance comprehension of the cytotoxic profiles associated with the two compounds and their combination, cellular proliferation was monitored over a 24-h period.

**Figure 5.**
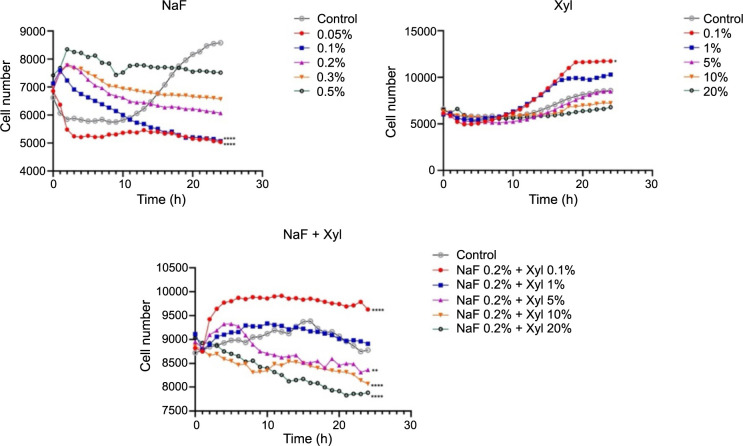
**Analysis of the effects of NaF, Xyl, and their combination (NaF + Xyl) on the proliferation of the HaCaT cells.** Statistical significance was calculated against the control group. *P < 0.05, **P < 0.01, ****P < 0.0001. NaF: Sodium fluoride; Xyl: Xylitol; HaCaT: Human keratinocytes.

In the context of HaCaT cells stimulated with NaF, a concentration-dependent reduction in the proliferation rate was noted, culminating in a plateau in cell count after 24 h. Notably, the highest NaF concentration (0.5%) precipitated a marked decrease in cell proliferation compared to the control group of unstimulated cells. Concerning the impact of Xyl on cell proliferation, the initial two examined concentrations (0.1% and 1%) elicited an augmentation in cell proliferation compared to control cells, while the concentration of 20% induced a proliferation inhibition. A comparable proliferative reaction was noted in the NaF and Xyl combination, leading to a decrease in cell proliferation, albeit not as pronounced as seen with Xyl administration alone (Figure 5).

**Figure 6.**
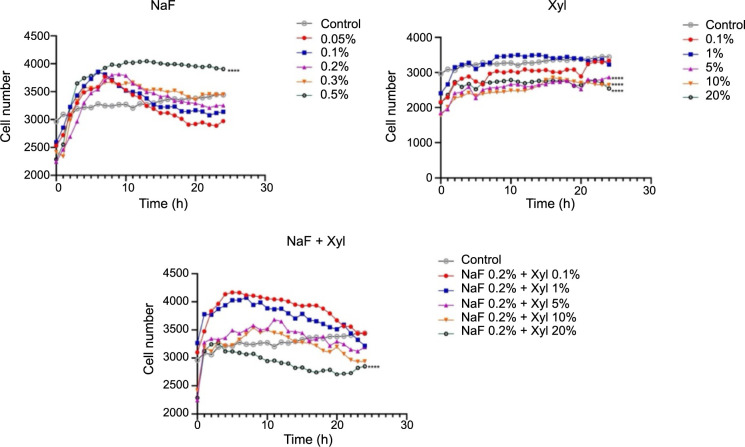
**Analysis of the effects of NaF, Xyl, and their combination (NaF + Xyl) on the proliferation of the SAOS-2 cells.** Statistical significance was calculated against the control group. ****P < 0.0001. NaF: Sodium fluoride; Xyl: Xylitol; SAOS-2: Osteosarcoma cells.

In SAOS-2 cells, NaF displayed a contrasting impact on cellular proliferation compared to that observed in HaCaT cells. In this scenario, lower concentrations exerted an inhibitory effect, while the highest concentration resulted in a noteworthy increase in cellular proliferation compared to the control. Conversely, Xyl demonstrated a concentration-dependent inhibitory effect on cellular proliferation, though to a lesser degree. The concurrent application of NaF and Xyl resulted in a decrease in cellular proliferation when compared to untreated cells, although this decline was not significant (Figure 6).

### Cellular morphology evaluation

The subsequent phase in the in vitro assessment of the two compounds and their combination entailed scrutinizing their impact on cellular morphology.

**Figure 7.**
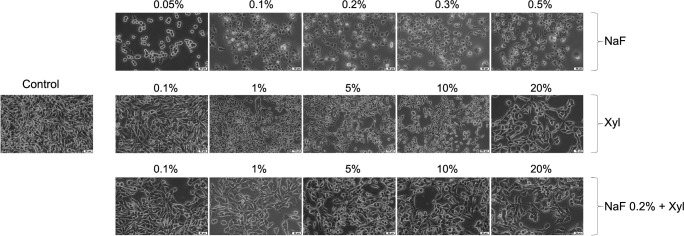
**Morphological alterations observed in HaCaT cells following 24 h of exposure to NaF, Xyl, and their combination (NaF + Xyl) in vitro.** The images were captured at a 20× magnification, accompanied by a scale bar representing 50 µm. HaCaT: Human keratinocytes; NaF: Sodium fluoride; Xyl: Xylitol.

Within HaCaT cells, exposure to NaF led to morphological alterations, including cellular rounding, detachment from the substrate, and disruption of intercellular connections, exhibiting an inversely proportional relationship with the applied concentration. The most prominent morphological changes occurred at the lowest concentration employed (0.05%), with milder alterations noted at higher concentrations. Conversely, Xyl induced dose-dependent modifications in cellular morphology. Concentrations up to 5% failed to induce noteworthy changes, maintaining a cellular morphology akin to that of unstimulated cells. Nevertheless, at a 20% concentration, morphological alterations suggestive of cellular death, such as cellular rounding and detachment from the substrate, were detected. The combined treatment of NaF and Xyl did not yield substantial morphological alterations; the discernible effect was a reduction in cellular confluence concomitant with an increase in the tested concentration (Figure 7).

**Figure 8.**
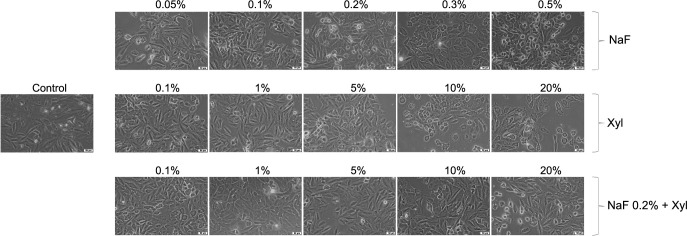
**Morphological alterations observed in SAOS-2 cells following 24 h of exposure to NaF, Xyl, and their combination (NaF + Xyl) in vitro.** The images were captured at a 20× magnification, accompanied by a scale bar representing 50 µm. SAOS-2: Osteosarcoma cells; NaF: Sodium fluoride; Xyl: Xylitol.

In the SAOS-2 cells, NaF instigated morphological alterations at the lowest concentration subjected to testing. In this instance, the observation of rounded and suspended cells indicated cellular demise. As the applied concentration escalated, the morphological changes exhibited a diminishing intensity, with cell shape and confluence converging toward those observed in control cells. In contrast, Xyl induced concentration-dependent morphological modifications. Lower concentrations in this context did not yield noteworthy deviations in comparison to control cells. However, the 20% concentration precipitated a reduction in cellular confluence and a modification in cell shape. The concurrent administration of NaF and Xyl did not result in significant changes, preserving cellular characteristics similar to those of control cells across all concentrations tested (Figure 8).

### Immunofluorescence

To comprehensively assess the impact on cellular morphology exerted by the tested compounds, an investigation into the aspects of nuclei and actin filaments was conducted after the application of sub-cytotoxic concentrations of NaF, Xyl, and their combination, employing the immunofluorescence method.

**Figure 9.**
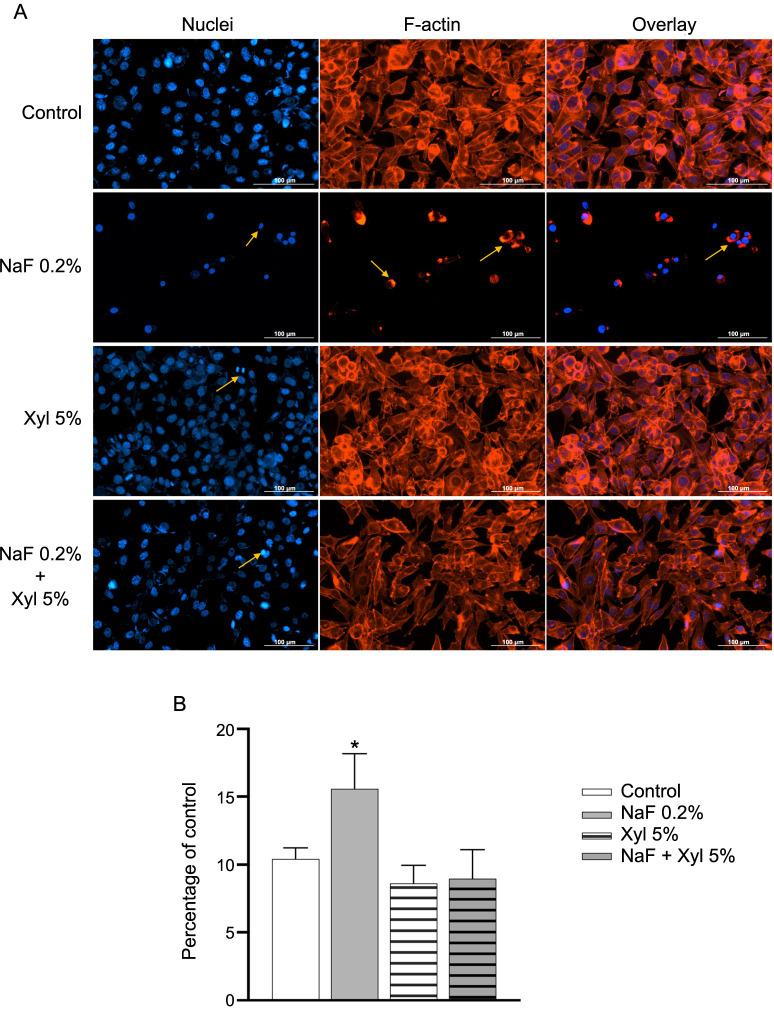
**The immunofluorescence technique employed to examine changes in nuclear and actin filaments structures in HaCaT cells, following treatment with sub-cytotoxic concentrations of NaF, Xyl, and their combination.** (A) Immunofluorescence staining illustrating the effects of NaF, Xyl, and their combination (NaF + Xyl) on actin filaments and nuclei in HaCaT cells, 24 h post-treatment. Nuclei were stained with DAPI (blue), and the actin filaments were stined with Texas Red™ -X Phalloidin (red). Yellow arrows highlight nuclear modifications and actin cytoskeleton remodeling. Scale bars represent 100 µm. (B) Analysis of the apoptotic index (AI) in HaCaT cells following exposure to NaF, Xyl, and NaF + Xyl. The AI is expressed as a percentage (%) normalized to the control and presented as mean ± standard deviation from three independent experiments. Statistical significance of the differences between control and treated cells were determined using one-way ANOVA, with subsequent Dunnett’s multiple comparison post-test. *P < 0.1. HaCaT: Human keratinocytes; NaF: Sodium fluoride; Xyl: Xylitol; DAPI: 4,6-diamidino-2-phenylindole; ANOVA: Analysis of variance; F-actin: Filamentous actin.

**Figure 10.**
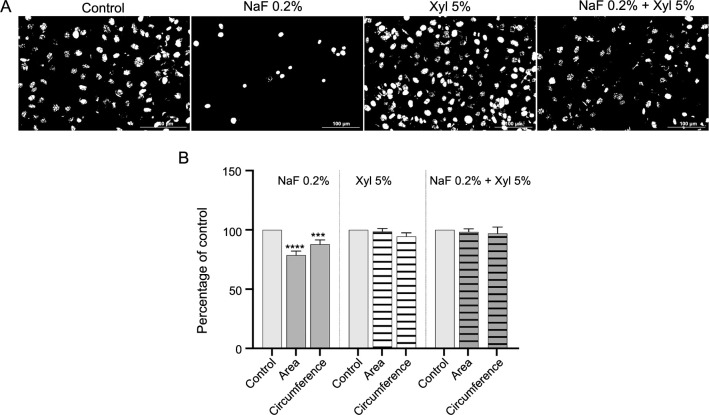
**Evaluation of the effects of NaF, Xyl, and their combination on nuclear area and circumference in HaCaT cells.** (A) Assessment of HaCaT cells nuclei after converting the images to 8-bit and applying the Make Binary function; (B) Comparatively to the control, the cells treated with 0.2% NaF exhibited a reduction in both nuclear area and circumference. ***P < 0.001; ****P < 0.0001. HaCaT: Human keratinocytes; NaF: Sodium fluoride; Xyl: Xylitol.

Within HaCaT cells, NaF induced noteworthy modifications in nuclear structure, resulting in a reduction in both size and number of nuclei in comparison to control cells. Additionally, a conspicuous condensation of chromatin was observed. In the context of actin filaments, NaF prompted their reorganization and induced robust condensation. On the contrary, Xyl at a concentration of 5% did not cause significant changes in the structure of nuclei and actin filaments, apart from the observed fragmentation at the nuclear level. Moreover, the combination of the two compounds elicited only a minor condensation of nuclear chromatin but otherwise left the analyzed cell organelle structures comparable to those in untreated cells (Figure 9A). Through the computation of the AI, it was determined that the exposure of HaCaT cells to NaF resulted in an elevation of its value, signifying cellular apoptosis. Conversely, in the context of Xyl and the combined treatment with NaF and Xyl (NaF + Xyl), the AI value exhibited no significant alteration (Figure 9B). When assessing the impact of these compounds on nuclear area and circumference, cells exposed to NaF showed a decrease in these measurements relative to control cells. Conversely, cells incubated with Xyl alone or in combination with NaF displayed no statistically significant effects on nuclear area and circumference (Figure 10A and 10B).

**Figure 11.**
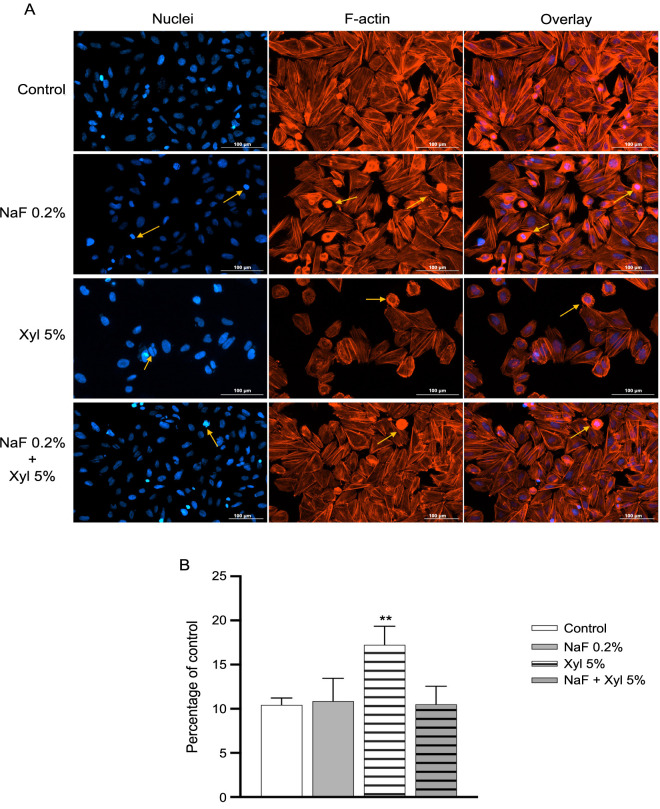
**The immunofluorescence technique employed to examine changes in nuclear and actin filaments structures in SAOS-2 cells, following treatment with sub-cytotoxic concentrations of NaF, Xyl, and their combination.** (A) Immunofluorescence staining illustrating the effects of NaF, Xyl, and their combination (NaF + Xyl) on actin filaments and nuclei in SAOS-2 cells, 24 h post-treatment. Nuclei were stained with DAPI (blue), and the actin filaments were stined with Texas Red™ -X Phalloidin (red). Yellow arrows highlight nuclear modifications and actin cytoskeleton remodeling. Scale bars represent 100 µm. (B) Analysis of the apoptotic index (AI) in SAOS-2 cells following exposure to NaF, Xyl, and NaF + Xyl. The AI is expressed as a percentage (%) normalized to the control and presented as mean ± standard deviation from three independent experiments. Statistical significance of the differences between control and treated cells were determined using one-way ANOVA, with subsequent Dunnett’s multiple comparison post-test. **P < 0.01. SAOS-2: Osteosarcoma cells; NaF: Sodium fluoride; Xyl: Xylitol; DAPI: 4,6-diamidino-2-phenylindole; ANOVA: Analysis of variance; F-actin: Filamentous actin.

At the cellular level in SAOS-2 cells, the application of NaF at a concentration of 0.2% elicited a subtle condensation of chromatin and prompted a reorganization and condensation of actin filaments. Nonetheless, the alterations in both the quantity and appearance of nuclei and actin filaments exhibited no significant deviation from that recorded for the control cells. In contrast, Xyl induced a series of profound modifications in nuclear structure, encompassing chromatin condensation, nuclear structure fragmentation, and the formation of apoptotic bodies. Concurrently, a reorganization of actin filaments manifested in the form of a peripheral ring. Concerning the combined exposure to NaF + Xyl, it resulted in mild condensation of nuclei and actin filaments, albeit less pronounced than the effects observed when Xyl was administered solely (Figure 11A). Furthermore, the computation of the AI underscored that SAOS-2 cells treated with Xyl exhibited an elevation in its value. In contrast, treatment with NaF alone and the combination of NaF + Xyl did not exert a statistically significant impact on the AI value (Figure 11B).

**Figure 12.**
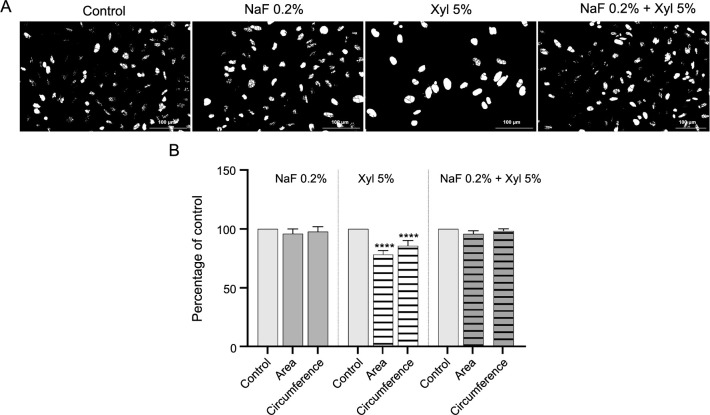
**Evaluation of the effects of NaF, Xyl, and their combination on nuclear area and circumference in SAOS-2 cells.** (A) Assessment of SAOS-2 cells nuclei after converting the images to 8-bit and applying the Make Binary function; (B) Comparatively to the control, the cells treated with 5% Xyl exhibited a reduction in both nuclear area and circumference. ****P < 0.0001. SAOS-2: Osteosarcoma cells; NaF: Sodium fluoride; Xyl: Xylitol.

Furthermore, through the assessment of nuclear area and circumference dimensions, it was noted that the treatment of SAOS-2 cells with Xyl culminated in a noteworthy decrease in these parameters. Meanwhile, the exposure of cells to NaF and to the combined administration of NaF with Xyl had no significant effect in comparison to controls (Figure 12A and 12B).

### Caspase-3/7 and 9 activity

The effects of NaF, Xyl, and their combination on caspases 3/7 and 9 activity were investigated following 24-h treatment period.

**Figure 13.**
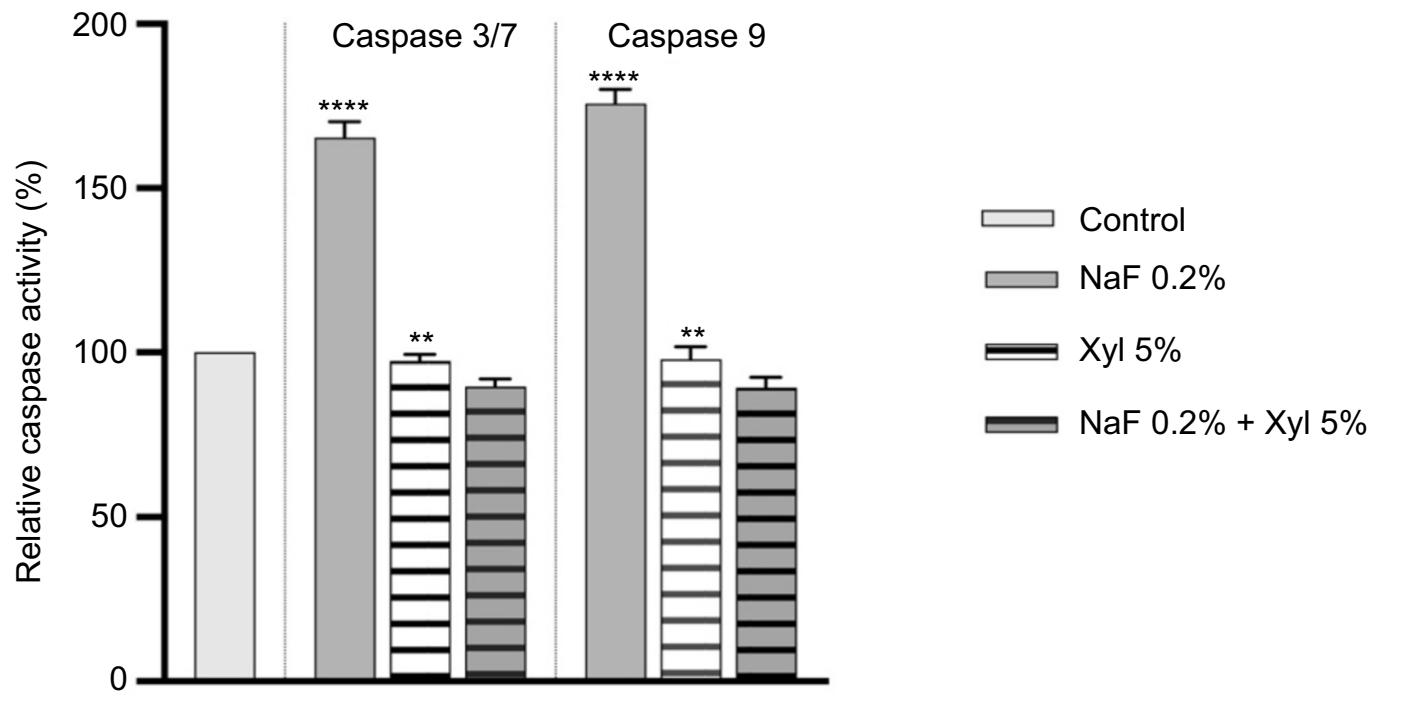
**Assessment of caspases 3/7 and 9 activity in HaCaT cells following 24-h exposure to NaF, Xyl, and their combination (NaF + Xyl).** Data are presented as the mean ± standard deviation from three independent experiments, normalized to the activity in the control group. Statistical analysis was performed using one-way ANOVA followed by Dunnett’s post hoc test to determine significant differences from the control group. **P < 0.01; ****P < 0.0001. HaCaT: Human keratinocytes; NaF: Sodium fluoride; Xyl: Xylitol; ANOVA: Analysis of variance.

In HaCaT cells, the application of NaF at 0.2% resulted in a substantial augmentation in the activity levels of both caspases. Likewise, Xyl induced an increase in caspase activity, though to a lesser degree. Conversely, the activity of caspases 3/7 and 9 remained insignificantly altered following a 24-h treatment with a combination of NaF at 0.2% and Xyl at 5% (Figure 13).

**Figure 14.**
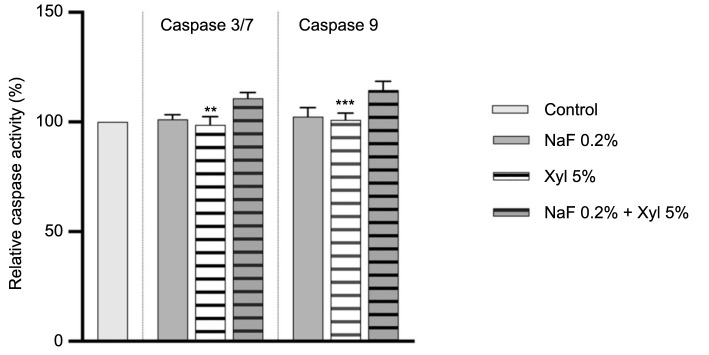
**Assessment of caspases 3/7 and 9 activity in SAOS-2 cells following 24-h exposure to NaF, Xyl, and their combination (NaF + Xyl).** Data are presented as the mean ± standard deviation from three independent experiments, normalized to the activity in the control group. Statistical analysis was performed using one-way ANOVA followed by Dunnett’s post hoc test to determine significant differences from the control group. **P < 0.01; ***P < 0.001. SAOS-2: Osteosarcoma cells; NaF: Sodium fluoride; Xyl: Xylitol; ANOVA: Analysis of variance.

In SAOS-2 cells, NaF exposure did not result in a noteworthy augmentation of caspases 3/7 and 9 activity. Conversely, a 24-h treatment with Xyl led to a slight decrease in their activity compared to control cells that were left untreated. Furthermore, the co-administration of NaF and Xyl exhibited no significant impact on the activity levels of the analyzed caspases (Figure 14).

### Gene expression ratio

Following the observed elevation in caspase activity, integral to the cellular apoptosis process, the subsequent investigation aimed to assess the expression of genes associated with cellular apoptosis.

**Figure 15.**
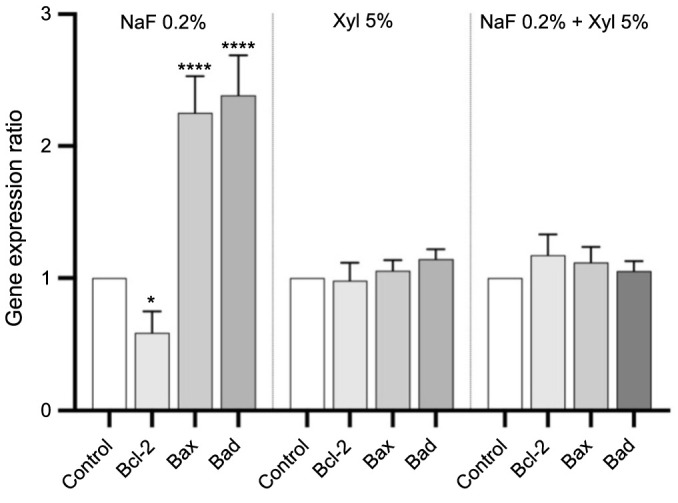
**Alterations in mRNA expression levels of pro-apoptotic (*Bax* and *Bad*) and anti-apoptotic (*Bcl-2*) markers in HaCaT cells following 24-h exposure to NaF, Xyl, and their combination (NaF + Xyl).** The mRNA expression levels were normalized to 18S expression and are presented as mean ± standard deviation from three independent experiments. Statistical significance was determined using one-way ANOVA followed by Dunnett’s post hoc test. *P < 0.1; ****P < 0.0001. mRNA: Messenger RNA; *Bax*: Bcl-2-associated X protein; *Bad*: Bcl-2-associated death promoter; *Bcl-2*: B-cell lymphoma 2; HaCaT: Human keratinocytes; NaF: Sodium fluoride; Xyl: Xylitol; ANOVA: Analysis of variance.

In HaCaT cells, NaF treatment induced a marked downregulation of the anti-apoptotic gene *Bcl-2*, accompanied by a notable upregulation of the pro-apoptotic genes *Bad* and *Bax*, compared to the control cells. In contrast, neither Xyl treatment nor the co-administration of NaF and Xyl led to substantial changes in the expression levels of these genes (Figure 15).

**Figure 16.**
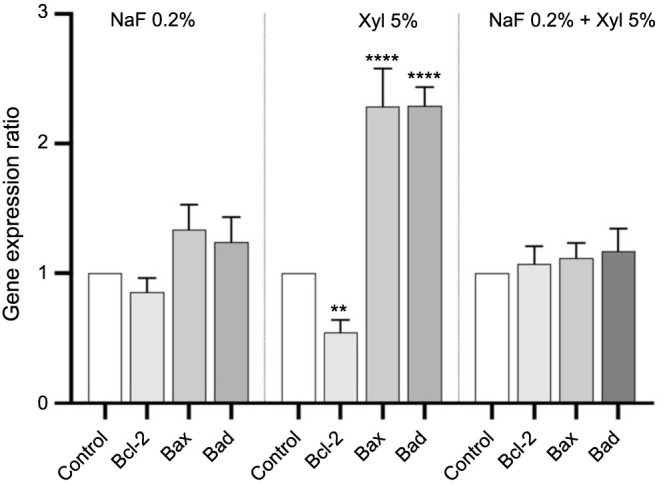
**Alterations in mRNA expression levels of pro-apoptotic (*Bax* and *Bad*) and anti-apoptotic (*Bcl-2*) markers in SAOS-2 cells following 24-h exposure to NaF, Xyl, and their combination (NaF + Xyl).** The mRNA expression levels were normalized to 18S expression and are presented as mean ± standard deviation from three independent experiments. Statistical significance was determined using one-way ANOVA followed by Dunnett’s post hoc test. **P < 0.01; ****P < 0.0001. mRNA: Messenger RNA; *Bax*: Bcl-2-associated X protein; *Bad*: Bcl-2-associated death promoter; *Bcl-2*: B-cell lymphoma 2; SAOS-2: Osteosarcoma cells; NaF: Sodium fluoride; Xyl: Xylitol; ANOVA: Analysis of variance.

In SAOS-2 cells, Xyl treatment elicited a significant downregulation of *Bcl*-2 expression while concurrently inducing a significant upregulation in the expression of pro-apoptotic genes. Nevertheless, neither NaF treatment nor the co-administration of NaF and Xyl produced significant changes in the gene expression compared to control cells (Figure 16).

### Hen’s egg test—chorioallantoic membrane (HET-CAM)

**Figure 17.**
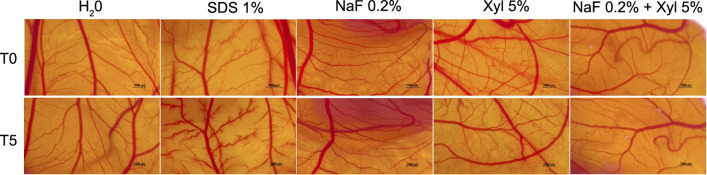
**Evaluation of the irritant potential of NaF, Xyl, and their combination (NaF + Xyl) utilizing the HET-CAM assay.** Figure displaying photographs of CAMs as viewed under a stereomicroscope, treated with a negative control (water), a positive control (1% SDS), and the test compounds. Images are captured at the initial time point (T0) and 5 min after application (T5). NaF: Sodium fluoride; Xyl: Xylitol; HET-CAM: Hen’s egg test-chorioallantoic membrane; SDS: Sodium dodecyl sulfate.

NaF, Xyl, and their combination were further assessed for potential irritative effects on the CAM, with concurrent assessments involving a negative control (water) and a positive control (1% SDS). The application of NaF to the vascular plexus elicited manifestations of vascular irritation, characterized by vascular lysis and microhemorrhage. In contrast, Xyl did not induce substantial alterations but rather induced a mild dilation of blood vessels. Moreover, the combined administration of NaF and Xyl demonstrated the least pronounced manifestations of vascular irritation on the CAM (Figure 17). Table 1 presents the IS obtained for each sample to quantify their irritative potential. As anticipated, the lowest IS was observed for water, while the 1% SDS produced the highest IS. The compounds under investigation showed IS of 1.4 for 0.2% NaF, 0.63 for 5% Xyl, and 0.48 for the combination of NaF and Xyl.

**Table 1:** The irritation scores for NaF, Xyl, and their combination (NaF + Xyl), alongside the onset times of hemorrhage, lysis, and coagulation

	**H_2_O**	**SDS 1%**	**NaF 0.2%**	**Xyl 5%**	**NaF 0.2% + Xyl 5%**
IS	0.07	19.93	1.40	0.63	0.48
tH	300	19	285	300	300
tL	300	17	269	290	295
tC	300	14	288	289	290

The time for tH, tL, and tC is represented in seconds. IS: Irritation score; NaF: Sodium fluoride; Xyl: Xylitol; SDS: Sodium dodecyl sulfate; tH: Time of hemorrhage; tL: Time of lysis; tC: Time of coagulation.

## Discussion

In alignment with the World Health Organization (WHO) recommendations, toothpaste is recommended to contain fluoride concentrations ranging from 1000–1500 ppm, while drinking water should contain 1.5 mg/L of it [20–22]. In spite of extensive research into the potential link between fluoride exposure and osteosarcoma incidence, the current evidence remains inconclusive [23, 24]. Moreover, Xyl-containing products are known to offer various therapeutic benefits [25]. The purpose of this study was to evaluate the influence of NaF, Xyl, and their combination on non-tumor keratinocyte cells (HaCaT) and osteosarcoma cells (SAOS-2).

To comprehend dental product safety, HaCaT cells can be used to study cytotoxicity and genotoxicity [26]. Additionally, SAOS-2 cells offer insights into the potential impact of dental products on bone tissue [27]. The concentrations of Xyl and NaF used in this study were determined based on their prevalence in dental products where they are found [28–30].

The cytotoxicity effects of NaF have been extensively studied in a wide variety of cell lines, which include renal cells, chondrocytes, osteoblasts, epithelial cells of the lung, and HaCaT. These studies showed dose- and time-dependent reductions in cell viability [31–36]. The effect of Xyl, on the other hand, was divergent, with lower doses enhancing cell proliferation while higher doses resulted in significant cell reductions. The effects of Xyl on HaCaT in counteracting NaF-induced toxicity have been demonstrated. Such interactions suggest potential benefits of incorporating Xyl in dental care products, like mouthwashes, not only to enhance enamel protection but also for its antimicrobial and anti-inflammatory properties [37–40].

SAOS-2 cells were subjected to tests with Xyl, NaF, and their combination. Previous research indicates that NaF increases SAOS-2 cell proliferation in a dose-dependent manner [41]. Similarly, another study on MG-63 osteosarcoma cells revealed that lower doses of NaF reduced cellular viability, whereas higher doses promoted cellular viability [42]. Furthermore, in populations consuming water with high fluoride levels, a correlation was observed between serum fluoride levels and osteosarcoma incidence [24]. Low doses of NaF may have cytotoxic effects through various mechanisms, such as changes in cell cycle regulation, DNA damage, and gene expression changes [43]. NaF may have biphasic and hormetic effects across different cell types, resulting in divergent responses to low and high doses of NaF [44, 45]. Xyl’s antitumor properties have been explored across various tumor cell lines. Tomonobu et al. [46] observed Xyl’s selective impact on pancreatic cancer cells by modulating glutathione levels. In a similar manner, Paark et al. demonstrated that Xyl exhibited antitumor activity in lung cancer cells (A549) via cytotoxicity mediated by autophagy [38]. In a study involving SAOS-2 cells, Murugan et al. [47] developed a nanohydroxyapatite reinforced with a poly (xyl sebacate) copolymer, which increased the generation of reactive oxygen species and induced cellular apoptosis. However, the direct antitumor effects of Xyl on SAOS-2 cells at clinically relevant concentrations have not yet been assessed. Based on its safety profile, this combination is recommended for dental formulations in oncological patients [48].

Apoptosis is characterized by distinct morphological changes at the cellular level, including cellular contraction, detachment from the substrate, and changes in nuclear and actin filament structures [49–52]. DAPI staining is a widely used technique in cell biology and microscopy for identifying nuclei due to its selective binding to the DNA. This versatile method facilitates various scientific investigations aimed at elucidating cellular processes and enhancing our understanding of biological phenomena [53, 54]. Additionally, staining with Texas Red-X Phalloidin provides insights into the dynamic cytoskeletal changes that occur during apoptosis [55]. Prado et al. have reported that DAPI staining revealed modifications in HaCaT cell nuclei induced by NaF treatment, corroborating previous observations of fluoride toxicity at the nuclear level [36, 56, 57]. Furthermore, NaF has been demonstrated to accelerate actin polymerization during apoptosis, contributing to cellular reorganization associated with this programmed cell death process [58–60].

The initiation of apoptosis is triggered by the activation of caspase 3/7, leading to nuclear condensation and rearrangement of actin filaments, which in turn causes blebbing and fragmentation of the cell membrane [61–63]. Caspase 9 plays a pivotal role in coordinating these processes through the release of cytochrome c from mitochondria [53, 64–66]. NaF has been shown to induce apoptosis in mouse kidneys by enhancing the activity of caspases 3, 8, and 9, as well as modulating caspase activity in mouse embryonic stem cells [67, 68]. Furthermore, Xyl has been found to induce apoptosis in tumor cells by modulating caspase-3 activity, which indicates that it has a pro-apoptotic effect [46].

The activity of caspases 3/7 and 9 correlates with elevated levels of the pro-apoptotic genes *Bax* and *Bad*, signaling the activation of the apoptotic pathway. Both *Bax* and *Bad* contribute to programmed cell death by affecting mitochondrial integrity and cytochrome c release [69]. The cell fate regulation is enhanced by coordinated molecular responses, reinforcing the robustness of the apoptotic program as well as improving our understanding of cellular death [70]. The findings of the current study reveal that NaF induces an upregulation in the expression of pro-apoptotic markers, specifically *Bax* and *Bad*, in HaCaT cells. Concurrently, a comparable increase in the expression of these markers was observed in SAOS-2 tumor cells when exposed to Xyl.

Our investigation extended beyond in vitro cytotoxicity assessments to evaluate the potential irritant effects of compounds using the HET-CAM method. This method is known for its predictive utility and serves as an alternative to animal testing [71]. Correlating the in vitro cytotoxicity findings with CAM irritancy assessments enhances safety evaluations and provides valuable information for therapeutic applications. This novel approach contributes to understanding the compound effects in this biological context, offering insights for future research.

The current study is subject to several limitations, including the choice of the in vitro model used. While in vitro models provide a controlled environment for studying cellular responses, they cannot fully replicate an in vivo environment. Hence, to comprehensively understand the potential biological mechanisms and cellular targets involved, further research is essential. Additionally, more studies are needed to ascertain the safety profiles of these compounds, their interactions, and their long-term effects. The findings of this study may also have clinical implications, particularly for the oncological field, since the compounds and their combination may serve as potential antitumor agents. Developing safe and effective therapeutic strategies also requires an understanding of the balance between their cytotoxic effects on tumor cells and their impact on healthy cells. It is important for clinicians to consider the potential side effects and toxicities associated with NaF and Xyl when developing treatment protocols.

## Conclusion

To conclude, this study highlights the dual effects of NaF and Xyl on cell behavior, including effects on cell viability, proliferation, apoptosis, and *Bcl-2* gene expression. While NaF exhibited an inversely proportional effect relative to the tested dose on cellular viability, proliferation, apoptosis, and the expression of *Bcl-2* family genes in both HaCaT and SAOS-2 cells, Xyl demonstrated dose-dependent cytotoxic effects accompanied by distinct morphological alterations. The combination of NaF and Xyl exhibited a nuanced response, influencing both the cell viability and morphology in SAOS-2 cells, suggesting potential therapeutic benefits for healthy cells and implications for oncological contexts. The interaction between NaF and Xyl unveils complex dynamics, offering insights that could steer future research endeavors and potential therapeutic strategies in the realm of cytotoxicity studies.

## Data Availability

The data presented in this study are available upon reasonable request from the corresponding author.
